# P-1828. Cost-effectiveness of Hepatitis C Screening in the Emergency Department

**DOI:** 10.1093/ofid/ofaf695.1997

**Published:** 2026-01-11

**Authors:** James I Barnes, Christopher T Buresh, Olivia P Hood, H Nina Kim

**Affiliations:** University of Washington, Seattle, Washington; University of Washington, Seattle, Washington; University of Washington, Seattle, Washington; University of Washington, Seattle, Washington

## Abstract

**Background:**

Chronic hepatitis C (HCV) is a leading cause of cirrhosis and hepatocellular carcinoma. An estimated 2.4 million people have chronic HCV in the US. Despite significant benefits of diagnosis due to highly effective treatment, it is estimated that 40% of patients with HCV are unaware of their infection. Multiple published studies have shown increasing screening in the emergency department (ED) substantially increases the number of patients diagnosed. We evaluated the cost-effectiveness of opt-out screening vs targeted screening (standard practice) for HCV in the ED.

**Figure:**
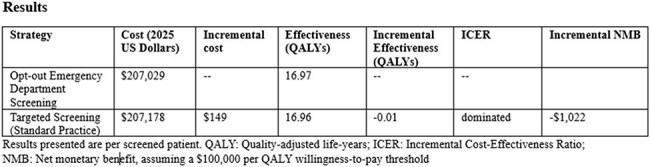
Cost-effectiveness Analysis Results

**Methods:**

A Markov model evaluated the cost-effectiveness of opt-out screening vs targeted screening for HCV in the ED from a US health-payer perspective over a lifetime horizon. Modeled clinical outcomes including progression through hepatic fibrosis states, development of hepatocellular carcinoma and mortality were calibrated to mirror published survival curves. We modeled costs of screening, linkage to care, therapies, liver transplant and other medical costs were modeled. Main outcomes were lifetime medical costs and quality-adjusted life-years (QALYs). Costs and benefits were discounted at 3% per year. Our base case modeled 6.9% of screened patients to be the HCV antibody (Ab) positive, with 54.9% of those with positive Ab HCV RNA positive, based on published literature. Linkage to care and prevalence parameters were based on published data and estimated from our institution’s experience. Multiple deterministic sensitivity analyses were performed.

**Results:**

Opt-out ED screening increased QALYs by 0.01 and resulted in cost savings of $149 per screened patient. Opt-out ED screening was, therefore, dominant (lower costs and higher QALYs) compared to standard practice (targeted screening). Dominance of the opt-out ED screening held across numerous variables in sensitivity analysis including a wide range of HCV prevalence (2-20% Ab positivity with corresponding 1-10% overall RNA positivity), with cost savings increasing with increasing prevalence.

**Conclusion:**

An ED opt-out HCV screening program is projected to provide modest gains in QALYs and modest cost-savings on a per-screened patient basis. An ED opt-out HCV screening program is projected to be cost-effective over targeted screening.

**Disclosures:**

James I. Barnes, MD, MS, Gilead: Grant/Research Support Christopher T. Buresh, MD, MPH, DTM&H, Gilead Life Sciences: Grant/Research Support Olivia P. Hood, MPH, CHES, Gilead Sciences: Grant/Research Support

